# Feasibility of conducting a pilot randomized trial of a mindfulness-based intervention among sheltered young adults experiencing homelessness

**DOI:** 10.3389/fpsyg.2025.1649664

**Published:** 2025-11-11

**Authors:** Diane Santa Maria, Paula Cuccaro, Erica Sibinga, Kimberly Bender, Ethel Jacko, Widumini Liyanage, Jennifer Jones, Stanley Cron

**Affiliations:** 1 Center for Nursing Research, Cizik School of Nursing, University of Texas Health Science Center at Houston, Houston, TX, United States; 2 Health Promotion and Behavioral Sciences, The University of Texas Health Science Center at Houston, Houston, TX, United States; 3 Department of Pediatrics, The Johns Hopkins University School of Medicine, Baltimore, MD, United States; 4 Graduate School of Social Work, University of Denver, Denver, CO, United States

**Keywords:** mindfulness, youth, young adults, homelessness, feasibility

## Abstract

**Purpose:**

Youth experiencing homelessness (YEH) are an underserved and difficult-to-reach population that experiences a disproportionate burden of trauma and stress compared to their housed peers. Prolonged trauma and stress can impact the development of negative emotions, reactive stress responses, and impulsive decision-making, which can lead to risk-taking behaviors. Growing research shows that Mindfulness-Based interventions (MBIs) can improve coping, impulsivity, emotion regulation, and executive function although no MBIs tailored for YEH have been tested.

**Methods:**

We conducted a pilot attention-control randomized trial to test the feasibility and acceptability of an adapted MBI, .*b4me (pronounced dot be for me)*, for youth living in a homeless shelter. *.b4me* is a five-session MBI adapted to address the unique considerations of YEH. We randomized youth to .*b4me* or the control condition, *Healthy Topics.* Each curriculum comprised 5 h-long group lessons delivered by trained facilitators. Pre- and post-lesson assessments were collected, as well as baseline, immediate-, 3- and 6-month post-follow-ups. Benchmarks for feasibility and acceptability were set *a priori*, and survey measures to assess emotional and psychological well-being were tested for feasibility and appropriateness of using these measures in future trials among this population and in a shelter setting.

**Results:**

The mean age of participants (*N* = 90) was 21.5 years old, with the majority identifying as male (62.2%), non-Hispanic (71.1%), black (50.0%), and heterosexual (55.6%). All *a priori* feasibility and acceptability benchmarks were surpassed and the reliability of most of the emotional and psychological well-being measures was confirmed.

**Conclusion:**

This study demonstrates that an MBI tailored for YEH, *.b4me*, is acceptable, and it is feasible to conduct a pilot attention control randomized trial with YEH living in a shelter despite major environmental obstacles.

## Introduction

Up to 4.2 million youth experience homelessness in the US (Morton et al., 2017). Among high school students across the US, 2.7% experience unstable housing according to the Youth Risk Behavior Survey (YRBS) (McKinnon, 2023). Those who do are more likely to engage in risk behaviors, including risky sexual behaviors, substance use, and suicide ideation and attempts. The challenges they face lead to a disparate burden of adverse health outcomes including death, suicide, substance use, overdose, pregnancy, HIV/STIs, and unmet mental health needs (Cauce et al., 2000; Doroshenko et al., 2012; Edidin et al., 2012; Grant et al., 2007; Kulik et al., 2011; Rosenthal et al., 2008; Smid et al., 2010). Youth experiencing homelessness (YEH) often have difficult family situations and histories of multiple traumas: poverty, physical, sexual, and emotional abuse (Gaetz, 2009). Moreover, YEH have high rates of parental addiction, psychiatric disorders, and criminal involvement that compound the trauma and instability experienced during childhood (Gaetz, 2004). The range of emotional and psychological challenges negatively impact their well-being, risk decision-making, emotion regulation, and coping skills. To this end, interventions aiming to increase YEH resilience must use a trauma-informed model that addresses their state of vulnerability, high levels of acute and chronic stress, unstable housing, trauma, and compromised executive functioning (Rew et al., 2001). The chronic stress of homelessness along with prevailing mood and anxiety disorders (Edidin et al., 2012) deflects attention away from disease prevention and healthy behaviors.

While the need for prevention and health promotion interventions tailored to the special considerations of YEH is undeniable, they continue to be understudied and underserved due to a chronically flawed sentiment that they are a challenging population to work with or study (Slesnick et al., 2000). To the contrary, YEH are able to be recruited and retained in intervention research and see improved outcomes when programs are tailored and relevant (Bender et al., 2015; Santa Maria et al., 2024).

Exposure to toxic stress, defined as an experience of strong, frequent, or prolonged stressful events (Shonkoff et al., 2012), is associated with a heightened risk of developmental or psychiatric disorders and health problems, including the development of chronic diseases (Briggs et al., 2013) and changes in brain structure and cognitive function, such as learning, working memory, and executive functioning tasks (Shonkoff et al., 2012). Interventions that simultaneously address both stress and risk behaviors may be more effective at risk prevention (Carmona et al., 2014). To this end, the American Academy of Pediatrics calls for programs to reduce toxic stress early in life to reduce the development of adult diseases and exacerbate health disparities (Shonkoff et al., 2012). Notably, mindfulness has been found to be protective for exposure to early life adversity (Whitaker et al., 2014).

Underlying psychosocial factors in youth should be addressed to support improvements in stress management, increased emotion regulation, and decreased impulsivity to optimize opportunities for behavioral change. Mindfulness-based interventions (MBI) teach mindfulness practices that can enhance self-regulation and self-observation through focused attention in the present moment (Goyal et al., 2014; Sibinga et al., 2011). MBIs that are trauma-informed and demonstrate acceptability may engage more young people than other service and intervention models (Brown and Bender, 2018) and have been found to improve mindful awareness and decrease psychological symptoms (Joss et al., 2024). Although the documentation on the benefits of mindfulness approaches on stress and anxiety reduction in adults is quite established (Goyal et al., 2014), there has been fewer studies conducted with youth, and even fewer with YEH (Grossman et al., 2004; Kabat-Zinn, 1982).

Evidence of the effectiveness of mindfulness approaches in adolescents shows decreased reactivity and increased mindful attention and awareness (Kuyken et al., 2013), enhanced self-regulation and coping among youth (Perry-Parrish et al., 2016), improved mental health and emotional control and reduced post-traumatic stress symptoms in urban youth (Sibinga et al., 2016), and well-being among youth in substance use programs and juvenile detention (Himelstein et al., 2015). Recent advances have led to the development of a neurodevelopmental framework that supports the potential for mindfulness as a self-regulation strategy particularly for young people with compromised self-regulation capacity, as is often the case with highly traumatized groups such as YEH (Kaunhoven and Dorjee, 2017). Reviews of MBIs (Burke, 2010) and meditation practices (Black et al., 2009) in youth suggest that these interventions may lead to reduced depression and anxiety (Zoogman et al., 2015). Other MBIs have found that mindfulness not only improves emotion regulation and well-being among substance-using youth (Himelstein et al., 2015) but it can also reduce symptoms of craving and withdrawal to aid in the treatment of substance use disorders (Lin et al., 2019). Despite the widespread dissemination of MBI strategies (Michalak and Heidenreich, 2018), little evidence exists to support its efficacy and no MBIs have been tailored specifically for YEH despite their high need for stress management, emotion regulation, and impulse control.

Many studies have suggested that MBIs have high levels of acceptability in urban, underserved youth (Mendelson et al., 2010), sexual and gender minority identifying younger adults (Seabra et al., 2024), and YEH (Bender et al., 2015; Grabbe et al., 2012; Santa Maria et al., 2020). According to a study conducted in HIV-positive and at-risk youth, out of those who attended any sessions, 79% participated in most sessions (Sibinga et al., 2011). A meta-analysis found significant effects on mindfulness, executive function, attention, depression, anxiety/stress, and risk behaviors among youth participating in an MBI compared to the control group (Klingbeil et al., 2017; Maynard et al., 2017; Semple et al., 2010; Zoogman et al., 2015). In particular, high acceptability was found among a school-based sample of youth between 12 and 16 years old for *.b* (“dot-be” which stands for stop and be)—the intervention adapted in the current study; this study also found decreased stress (*p* = 0.05), with the amount of practice being associated with reduced stress (*p* = 0.03) (Kuyken et al., 2013). Other significant benefits associated with mindfulness, depression, and anxiety have also been found among in randomized controlled trials (Sibinga et al., 2016; Zoogman et al., 2015).

Among limited evidence testing of an MBI with unhoused young people (*N* = 97), one prior study found high intervention engagement, uptake of the practices, and significant improvement in observational skills (Bender et al., 2015). Although the intervention improved attention to external and internal stimuli in youth, the findings suggested that tailoring would be beneficial to meet the unique needs of YEH. In a smaller quasi-experimental study, an MBI was delivered to YEH over 8 weeks (Burke, 2010; Viafora et al., 2015). Although the differences were nonsignificant, YEH reported improved emotional well-being, a greater likelihood of using mindfulness practices at school to deal with difficult emotions, and a greater likelihood of recommending mindfulness to their friends. Many YEH-serving organizations are utilizing mindfulness strategies despite the lack of rigorous data from randomized trials among YEH. Despite promising preliminary findings and strong rationale that interventions for highly stressed populations need to address stress as an antecedent of risk behavior (Brown et al., 2007; Chiesa et al., 2013; Teper and Inzlicht, 2013), well-designed trials are needed to assess if MBIs tailored to YEH are feasible and acceptable.

Building on the promising results of the *.b* pilot study (Santa Maria et al., 2023) the goal of this study was to conduct a feasibility pilot attention control randomized trial of an adapted MBI, *.b4me (pronounced dot be for me)*, among YEH ages 18–25 living in a shelter. *.b4me* was adapted using ADAPT-ITT in collaboration with YEH and health and social services providers (Santa Maria et al., 2023). *.b4me*, was adapted from the original *.b* curriculum during the first phase of the study and is described in a separate publication (Santa Maria et al., 2023). Key curriculum concepts and strategies include paying attention, fostering curiosity, self-compassion, understanding rumination and catastrophizing, staying in the present, recognizing thoughts as separate from self, responding instead of reacting, understanding stress and its impact, accepting negative and positive experiences, moving mindfully, and using mindfulness in everyday life. In summary, adaptations to the curriculum and delivery modality were made to approximate the average length of stay in the shelter, integrate trauma-informed approaches, increase the diversity of images used by race/ethnicity, age, sexual orientation, and gender identity, and increase the relevance of the audio-visual components to modern youth culture.

## Methods

This research study was approved by the Committee for the Protection of Human Subjects. A data safety monitoring board was assembled and met quarterly after enrollment began to monitor participant recruitment, accrual and retention rates, and to assess for any adverse events or protocol deviations.

### Participants

Ninety YEH aged 18–25 were recruited using convenience sampling from the largest shelter serving unaccompanied young adults in one large metropolitan area in the U.S. South. The shelter offers a temporary place to stay, a transitional living program, comprehensive case management, life skills, educational/vocational training, and health and mental healthcare. Eligibility criteria included being 18–25 years old, able to speak, read, and understand English, expected to stay at the shelter for at least 3 weeks (the duration of the intervention), and could read English (scored over four on the Rapid Estimate of Adult Literacy in Medicine [REALM] health literacy assessment) at the time of recruitment. This protocol for low literacy has been used successfully in our previous studies with homeless youth and did not result in any exclusions. Participants were excluded if there was concern that they were under the influence of substances or experiencing heightened mental health symptoms. In these cases, prospective youth were asked to come back at a later time to determine if they were eligible to participate.

### Procedure

Study staff visited the shelter about 3 days a week and provided a brief study description during meetings and announcements. Interested youth spoke with staff individually to learn more about the study and be screened for eligibility. Research staff utilized a three-step process for enrollment over the course of several days. Participants were considered officially enrolled in the study if they completed the consent and baseline survey and attended at least one session of the intervention or control sessions. Enrolled participants were issued a smartphone when they attended the third pre-study visit. This tiered enrollment process has been implemented in previous studies to provide ample time to consider participation, to avoid drop-out due to leaving the shelter quickly, and to ensure responsible use of study resources.

**Figure 1:**
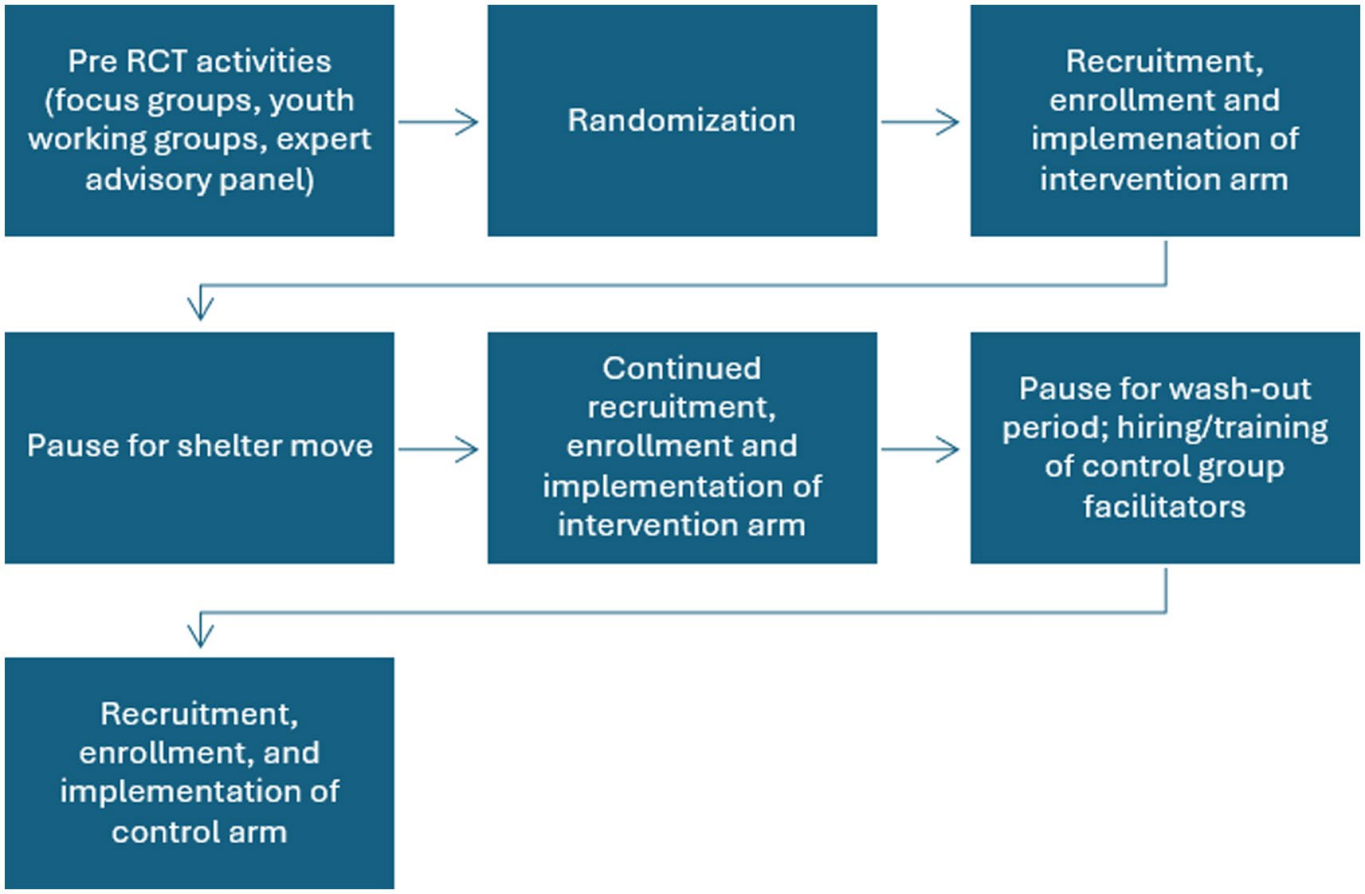
Study flow.

The order of the conditions, not the individual, was randomized in a one-stepped wedge design with the intervention group being offered first followed by the control condition. During the 17 months of recruitment, 5 months were not active due to the shelter being demolished and relocated to a temporary facility and the planned washout period (3 months) between recruitment into the two groups. Once the shelter had reached at least a 50% resident turnover, youth were recruited for the control arm. This was assessed by viewing the resident roster to determine when there was less than 50% of the same residents at the time of enrollment (Figure 1).

The intervention and control condition sessions were held on-site at the shelter 2–4 times a week in the morning, evening after dinner, and on various days of the week. Sessions were offered multiple times to maximize accessibility given the heterogeneity of schedules among sheltered youth. Attendees completed brief pre- and post-assessments at each session and received a $10 gift card for each session attended. Participants also completed follow-up surveys at immediate post, 3-, and 6 months, for which they received a $15 and $20 gift card, respectively. Surveys were administered in person using iPads, and follow-up surveys were administered in person or remotely by sending a link to the study-issued phone to access the survey. Individual exit interviews (*N* = 18) were conducted after implementation of the intervention or control curricula to collect feedback on study procedures, barriers and facilitators to session attendance, and acceptability of program content and delivery modality. After 18 interviews, no new themes were emerging and saturation was reached.

Three interventionists completed an eight-week mindfulness-based stress reduction course, attended the original *.b* curriculum training (5-day course), and maintained a personal mindfulness practice for at least 6 months prior to being prepared to deliver *.b4me* as part of this study. Upon completion of the background training, both interventionists received an orientation of the adapted curriculum *.b4me* and an overview of all study procedures to prepare for implementation.

The health education program *Healthy Topics* (HT) adapted from the Glencoe Health Curriculum (McGraw Hill) was modified from its original eight-session format to match the intervention condition delivery period and serve as an active control condition. This curriculum has served as the control condition to similar studies with youth populations (Mendelson et al., 2020; Sibinga et al., 2013; Sibinga et al., 2014). HT was matched to *.b4me* across session frequency, length, group size, location, timing, and instruction modalities. Topics covered in HT included physical activity, nutrition, managing weight, understanding adolescence, personal care, and avoiding tobacco, alcohol, and drugs. The HT program was led by two trained instructors with backgrounds in health education and experience working with adolescents to avoid contamination of mindfulness strategies across the control condition.

### Measures

Feasibility and acceptability measures were collected to determine if *a priori* benchmarks were met (Table 1). Recruitment, treatment adherence, and retention benchmarks were chosen considering the highly variable lengths of stay for young adults in a shelter. The study aimed to enroll 50% of all screened and eligible participants, expose at least 50% of participants to three out of five group sessions, and retain at least 50% of participants at the 3- and 6- months post-follow-up with a group retention differential less than 15%, attain less than 50% of data loss, and achieve over 6 on the credibility and expectancy questionnaire scale (Devilly and Borkovec, 2000). Setting a minimum of three sessions for the participation benchmark was informed by previous studies that found significant intervention effects even without exposure to an entire intervention curriculum (Biegel et al., 2009; Himelstein et al., 2012; Tucker et al., 2017).

**Table 1:** Feasibility and acceptability benchmarks.

Construct	Measure	Benchmarks
Recruitment feasibility	Number screened & enrolled/month;	Enroll 50% of screened and eligible participants
Treatment adherence	Attend all sessions	At least 50% of participants will attend at least 3 of 5 scheduled group sessions
Retention feasibility	3- and 6-month follow-up survey completion; compare intervention vs. control group	At least 50% retained at 3 and 6-months; <15% group retention differential
Completeness of assessment data	Data from surveys and exit interviews are complete and intact	Less than 50% data loss or incompleteness
Acceptability	Credibility/expectancy questionnaire	Average score of >6

Participants completed baseline and follow-up surveys immediately post-intervention and at 3- and 6-months post-intervention. In addition, participants completed very brief assessments immediately before and after each session of the intervention or control curriculum. The baseline survey asked about demographics including age, race, ethnicity, gender identity, sexual orientation, employment, and education level. A history of homelessness, involvement in the foster care and juvenile justice systems, and experiences of adverse childhood events were also collected.

Measures chosen to assess emotional and psychological well-being were informed by the study team, expert advisors, and cognitive interviews with the youth working group. The final baseline, immediate-, 3-, and 6-month follow-up surveys included scales to assess loneliness, stress (PSS), depression (PHQ-9), anxiety (GAD), emotional distress, anger, sleep disturbance/impairment, risk propensity, coping, emotion regulation, mindfulness, self-compassion, psychosocial vulnerability, social connectedness, resilience, social isolation, self-efficacy, executive function, and risk behaviors (see Table 2). The surveys administered pre- and post-session contained the same scales to assess mindfulness, emotion regulation, and anger, a shortened scale to measure stress, and a scale to measure positive and negative affect. The semi-structured exit interview guide was developed to assess the overall experience of participating in the study, expectations and perceived outcomes of the program, facilitators and barriers of attendance, experience with the session facilitators, acceptability of program content and delivery modality, and feedback on outcome measures and survey format and length.

**Table 2:** Outcome measures for pilot testing.

Construct	Scales	Psychometrics
Measures at baseline, immediate, and 3-, and 6-months		
Loneliness	NIH Toolbox Item Bank v2.0—Loneliness (Ages 18+)—Fixed Form	NA
Stress	Perceived Stress Scale (Cohen et al., 1983)	*α* = 0.91
Depression	Patient Health Questionnaire-9 (Kroenke et al., 2016)	*α* = 0.82–0.85
Anxiety	Generalized Anxiety Disorder (Kroenke et al., 2016)	*α* = 0.85–0.88
Distress	PROMIS Item Bank v.1.0—Emotional Distress—Anxiety- Short Form 7a (Wilford et al., 2018)	*α* = 0.95–0.96
Anger	PROMIS Item Bank v1.1—Anger	NA
Sleep quality	PROMIS Item Bank v1.0—Sleep Disturbance—Short Form 8b; PROMIS Item Bank v. 1.0—Sleep-Related Impairment—Short Form 8a (Forrest et al., 2018)	*α* = 0.90–0.92
Risk seeking	Risk Propensity Scale (Meertens and Lion, 2008)	*α* = 0.77
Coping	Brief COPE (Carver, 1997)	*α* = 0.500–0.90
Emotion regulation	Difficulties in Emotion Regulation Scale (DERS) (Kaufman et al., 2016)	*α* = 0.81–0.99^*^
Mindfulness	Child and Adolescent Mindfulness Measure (de Bruin et al., 2014)	*α* = 0.82^**^
Self-Compassion	Self-Compassion Scale (Raes et al., 2011)	*α* = 0.91^**^
Vulnerability	Psychological Vulnerability Scale (Sinclair and Wallston, 1999)	*α* = 0.71–0.86
Social Connectedness	Social Connectedness Scale; Interpersonal Support Evaluation (ISEL-12) (Lee and Robbins, 1995); Social Isolation –Short Form 4a (Hahn et al., 2014)	*α* = 0.91, 0.77^*^
Resilience	Resilience Portfolio Questionnaire manual: Scales for youth (Self-reliance) (Hamby et al., 2018)	*α* = 0.81
Self-Efficacy	General Self-Efficacy Scale (GSE) (Luszczynska et al., 2005)	*α* = 0.75–0.91
Executive function	The Amsterdam Executive Function Inventory (AEFI) (Van der Elst et al., 2012)	*α* = 0.60–0.65^**^
Sexual activity and drug use	Youth Risk Behavior Survey Items on sexual activity, condom use, and substance use	NA
Pre- and post-session measures for pilot testing		
Affect	Positive and Negative Affect Scale (Watson et al., 1988)	*α* = 0.78^**^
Mindfulness	Child and Adolescent Mindfulness Measure (de Bruin et al., 2014)	*α* = 0.82^**^
Emotion regulation	Difficulties in Emotion Regulation Scale (DERS) (Kaufman et al., 2016)	*α* = 0.81–0.99^*^
Anger	PROMIS Item Bank v1.1—Anger	NA
Stress	Perceived Stress Scale (Cohen et al., 1983)	*α* = 0.91^**^

* Tested among youth.

** Tested among YEH.

### Data analysis

To analyze quantitative data, frequencies and percentages were calculated to determine the rates of recruitment, retention, attendance, and data completeness. Emotional and psychological well-being measures were tested for reliability. As a feasibility study, changes in these measures are outside the scope of this study and not reported here.

Exit interviews were recorded to collect qualitative data and the audio files were transcribed by a third-party HIPAA compliant service. Codes were developed to capture the content discussed. Transcriptions were coded by trained team members who were not directly involved in the intervention delivery or data collection and were grouped according to theme. The investigators summarized themes and identified quotes that exemplified each theme.

## Results

### Recruitment

From March 2022 to August 2023, 121 individuals were screened, and 118 consented to reach the goal end sample size of 90 participants (45 intervention and 45 control). Youth at the shelter showed high interest in the program. As part of the study design, participants were provided with a study-issued phone to enhance follow-up and retention efforts.

### Sample characteristics

Participants were predominantly male (62.2%), non-Hispanic (71.1%), Black (50.0%), and heterosexual (55.6%). The mean age at enrollment was 21.5 and the mean age at first homelessness was 17.7. There were statistical differences in age (*p* = 0.029) and age at first homelessness (*p* = 0.022) in baseline characteristics between groups (Table 3). However, these differences between the groups were not deemed to be practically significant.

**Table 3:** Sample characteristics (*N* = 90).

Characteristic	Arm		Total (*N* = 90)	*p*-value
	Control (*n* = 45)	Intervention (*n* = 45)		
Age, y				0.029^1^
*N*	45	45	90	
Mean (SD)	21.9 (1.81)	21.1 (1.86)	21.5 (1.88)	
Age at first homelessness, y				0.022^1^
N	44	44	88	
Mean (SD)	18.6 (3.34)	16.8 (3.97)	17.7 (3.76)	
Gender identity, *n* (%)				0.065^2^
Male	27 (60.0)	29 (64.4)	56 (62.2)	
Female	17 (37.8)	10 (22.2)	27 (30.0)	
Transgender or another gender identity	1 (2.2)	6 (13.3)	7 (7.8)	
Ethnicity, *n* (%)				1.00^2^
Hispanic	13 (28.9)	13 (28.9)	26 (28.9)	
Non-Hispanic	32 (71.1)	32 (71.1)	64 (71.1)	
Race, n (%)				0.054^2^
Black	27 (60.0)	18 (40.0)	45 (50.0)	
White	4 (8.9)	12 (26.7)	16 (17.8)	
Multiple races and other	14 (31.1)	15 (33.3)	29 (32.2)	
Sexual orientation, *n* (%)				0.092^2^
Heterosexual	29 (64.4)	21 (46.7)	50 (55.6)	
Pansexual, asexual, questioning	10 (22.2)	8 (17.8)	18 (20.0)	
Bisexual	4 (8.9)	8 (17.8)	12 (13.3)	
Gay/Lesbian	2 (4.4)	8 (17.8)	10 (11.1)	

1 Student’s *t*-test *p*-value.

2 Chi-Square *p*-value.

### Intervention arm

Implementation of the intervention began in April 2022. Staff were onsite 66 times over 6 months. Due to the demolition of the shelter, the residents moved to a temporary site in October, 2022, after the implementation of the intervention arm commenced. To allow time for staff and residents to acclimate to their new environment, implementation of the intervention was paused in October, 2022 and resumed in November, 2022. Sessions were provided in semi-private spaces such as a meeting room, library, cafeteria, or shared living space in the mornings and evenings 2–4 times a week depending on shelter and trained facilitator schedule. Participants were not required to attend all sessions within a specific time range to maximize flexibility and participant access to the intervention. Implementation of the intervention arm ended in January, 2023. Thirty-five participants (77.8%) completed over half of the intervention curriculum with 27 (60%) completing the entire curriculum. While three facilitators were trained to deliver the intervention curriculum, one main facilitator delivered most of the sessions due to relocation and availability changes due to the pandemic.

### Control arm

The control condition, *Healthy Topics* was implemented entirely at the temporary shelter between April, 2023 and August, 2023 with staff being onsite 53 times over five consecutive months. Participants in the control arm were not required to attend all classes within a specific time range, similar to the intervention arm. Forty-one participants (91.1%) completed over half of the control curriculum with 24 (53.3%) completing the entire curriculum. Two trained facilitators delivered sessions equally.

### Retention

Follow-up data collection was challenging due to the transient nature of the participants. Twenty-five youth left the shelter during the study period. While youth were able to return to the shelter to participate in the sessions, transportation was a prohibitive factor for those housed through vouchers in apartments that were far from the shelter, or staying with family or friends who lived out of town. Aside from leaving the shelter, additional reasons for not completing the sessions may have been finding a job, enrolling in school or vocational program, or loss of interest. Further, the immediate post-survey was designed to be administered immediately after the last session. However, youth (*n* = 18) did not participate in the last session. Youth (*n* = 3) also left the shelter but returned months later for 3- and 6-month follow-ups. Despite these common realities of the lived experience of youth who are unhoused, benchmarks for retention were met (Table 4).

**Table 4:** Immediate-, 3-, and 6-month post-intervention follow-up rates.

Milestone	Intervention (%)	Control (%)	Total (%)
Immediate post	37 (82.22)	36 (80.00)	73 (81.11)
3-Month post	31 (68.89)	28 (62.22)	59 (65.56)
6-Month post	25 (55.56)	24 (53.33)	49 (54.44)

### Data completeness

The overall percentage of missing survey items across all surveys being 0.43% (median) for the control participants, 0.00% (median) for the intervention participants, and 0.43% (median) for both groups, which equated to 1 out of 235 items.

### Acceptability

The Credibility/Expectancy (*n* = 33; mean = 7.3; Std Dev = 1.96) score (CEQ) for the intervention group indicated that the adapted curriculum was acceptable. An acceptable CEQ score for the control group (*n* = 32; mean = 6.3; Std Dev = 1.96) score (CEQ) indicates the control program was also acceptable for this sample. A summary of feasibility and acceptability outcomes are outlined in Table 5.

**Table 5:** Feasibility and acceptability benchmarks and outcomes.

Construct	Benchmarks	Intervention group	Control group	Overall results
Recruitment feasibility	Enroll 50% of screened and eligible participants	Out of 67 screened and eligible, 45 were enrolled (67.2%)	Out of 54 screened and eligible, 45 were enrolled (83.3%)	Enrolled 74% of the screened and eligible participants
Treatment adherence	At least 50% of participants in each cohort will attend at least 3 of 5 scheduled group sessions	77.8% (*n* = 35) attended at least 3 of 5 group sessions	91.1% (*n* = 41) attended at least 3 of 5 group sessions	84% (*n* = 76) of participants attended at least 3 of 5 group sessions
Retention feasibility	At least 50% of participants retained at 3 and 6-mo; <15% group retention differential	68.9% (*n* = 31) retained at 3 month follow up 55.6% (*n* = 25) retained at 6 month follow up	62.2% (*n* = 28) retained at 3 month follow up 53.3% (*n* = 24) retained at 6 month follow up	65.6% (*n* = 59) retained at 3 month follow up54.4% (*n* = 49) retained at 6 month follow up 7 and 2% retention differential at 3- and 6-months, respectively
Completeness of assessment data	Less than 50% data loss or incompleteness	Median = 0.00%	Median = 0.43%	Median = 0.43%
Acceptability	Average score of >6	CEQ score mean = 7.3 (n = 33; Std Dev = 1.96)	CEQ score mean = 6.3 (*n* = 32; Std Dev = 2.50)	CEQ score mean = 6.8 (*n* = 65; Std Dev = 2.29)

### Outcome measures for pilot testing

To assess the reliability of outcome measures in the baseline and follow-up surveys, Cronbach alpha scores were calculated for all scales and ranged from *α* = 0.34–0.97 (Table 6). All outcomes were deemed to have acceptable reliability except self-compassion and risk-seeking measures. To assess the reliability of outcome measures in the pre-and post-session surveys, Cronbach alpha scores were calculated for all scales and ranged from α = 0.22–0.96. All outcomes were deemed to have acceptable reliability except the abbreviated stress scale. There were no adverse events or protocol deviations during this study.

**Table 6:** Reliability of outcome measurements.

Construct	Scales tested	Pilot alpha
Baseline, immediate, and 3-, and 6-months		
Loneliness	NIH Toolbox Item Bank v2.0—Loneliness (Ages 18+)—Fixed Form	*α* = 0.93
Stress	Perceived Stress Scale (Cohen et al., 1983)	*α* = 0.72
Depression	Patient Health Questionnaire-9 (Kroenke et al., 2016)	*α* = 0.92
Anxiety	Generalized Anxiety Disorder (Kroenke et al., 2016)	*α* = 0.93
Distress	PROMIS Item Bank v.1.0—Emotional Distress—Anxiety—Short Form 7a (Wilford et al., 2018)	*α* = 0.94
Anger	PROMIS Item Bank v1.1—Anger	*α* = 0.97
Sleep quality	PROMIS Item Bank v1.0—Sleep Disturbance—Short Form 8b; PROMIS Item Bank v.1.0—Sleep-Related Impairment—Short Form 8a (Forrest et al., 2018)	*α* = 0.83 *α* = 0.83
Risk seeking	Risk Propensity Scale (Meertens and Lion, 2008)	*α* = 0.62
Coping	Brief COPE (Carver, 1997)	*α* = 0.91 *α* = 0.88 *α* = 0.80
Emotion regulation	Difficulties in Emotion Regulation Scale (DERS) (Kaufman et al., 2016)	*α* = 0.93
Mindfulness	Child and Adolescent Mindfulness Measure (de Bruin et al., 2014)	*α* = 0.94
Self-Compassion	Self-Compassion Scale (Raes et al., 2011)	*α* = 0.34
Vulnerability	Psychological Vulnerability Scale (Sinclair and Wallston, 1999)	*α* = 0.86
Social Connectedness	Social Connectedness Scale; Interpersonal Support Evaluation (ISEL-12) (Lee and Robbins, 1995); Social Isolation—Short Form 4a (Hahn et al., 2014)	*α* = 0.95 *α* = 0.86
Resilience	Resilience Portfolio Questionnaire manual: Scales for youth (Self-reliance) (Hamby et al., 2018)	*α* = 0.88
Self-Efficacy	General Self-Efficacy Scale (GSE) (Luszczynska et al., 2005)	*α* = 0.94
Executive function	The Amsterdam Executive Function Inventory (AEFI) (Van der Elst et al., 2012)	*α* = 0.72
Pre- and post-session measures		
Affect	Positive and Negative Affect Scale (Watson et al., 1988)	*α* = 0.90 (+) *α* = 0.92 (−)
Mindfulness	Child and Adolescent Mindfulness Measure (de Bruin et al., 2014)	*α* = 0.93
Emotion regulation	Difficulties in Emotion Regulation Scale (DERS) (Kaufman et al., 2016)	*α* = 0.93
Anger	PROMIS Item Bank v1.1—Anger	*α* = 0.96
Stress	Perceived Stress Scale (Cohen et al., 1983)	*α* = 0.22

### Qualitative findings

Overall, the youth described the usefulness of *.b4me* and how it helped them with emotion regulation, mindfulness, and coping. Based on the responses in the exit interviews, youth felt that the sessions had benefitted them in many ways. *“Well, it’s very interesting. It helped me cope with a lot of things.”* Several participants spoke about how the mindfulness practices learned in the intervention helped to clear their minds. One participant mentioned regaining control over their thoughts and said, “*Honestly, it was kind of eye-opening to the fact that my brain could work a certain way, and I could learn to tame it.”* Another participant described the intervention as “*learning to help your brain stop, going back into that primal mode that it was set in for years.”* One youth felt that there were also other potential benefits for others. They said, “*I feel like people with anxiety disorder could benefit too, just learning how to, like, get a hold on their brain.”* When asked the purpose of the study, one participant believed that it was *“to spread awareness of your brain and how it works and how to fix what has been wired into it*.” In general, the study sessions seemed to be positively received. As stated by one of the participants, *“It helped me out throughout the whole session,… before I even got into the sessions and stuff, I just felt angry most of the time. You know, it helped me learn new skills, how to cope with it, and…better myself.”* Additional themes are summarized in Table 7.

**Table 7:** Themes and exemplars from participant feedback.

Feedback on session activities	*“So, in the videos, they made a lot of metaphors, and like the visuals…they made like comedic comparisons, which I think made it really easy to grasp the concepts. And all of the activities that we did, you know, they were very simple. So, you can make those clear connections between the two things she (facilitator) was talking about.”*
Feedback on session activities	“*Those (discussions) were very engaging, and I found that it helped me process what she (facilitator) had said before, earlier, better.”*
Feedback on session activities	“*Honestly, the walking [meditation] one (was the least favorite) because I felt like I was going to trip.”*
Continued use of new mindfulness skills	*“I was genuinely finding a lot of use using the .b* [pause and be] *method and meditating in general. I still do that just to kind of corral my thoughts,”*
Continued use of new mindfulness skills	“*I would like practice in my room here. Whenever I would go to sleep, I would just like try to focus on where I was laying in my bed and just focusing on my breathing. Because like focusing on my breathing, it’s been really helpful*.”
Engagement facilitators	“*I found it to be really convenient because it was on-site, so I did not have any trouble going.”*
Engagement facilitators	*“It was just excellent how they just helped out the kids, motivated them to go to the meetings. You know, we will pay attention and see how hard they put an effort. So, you know, we would pay attention, pay attention hard, you know. And it helps us to be better.*”
Engagement facilitators	“*I really liked the amount of sessions. I think for most of the people here, it might be a little long, but I think that’s what you need to really capture how much there was to the program. I do not think there’s a way to consolidate it without losing something in the process.”*
Engagement facilitators	“*I thought that the incentives were really nice. Like, there was like food in the morning to eat. It was really nice that they provided those (hygiene kits) for us if we needed them.”*
Suggestions for improvement	*“I think they were repetitive, but I feel like they were needed.”*
Suggestions for improvement	“*I think the length of the questions—I think that’s what really… just the length.”*

There were various activities, including videos, worksheets, discussions, and various mindfulness/meditation practices. Participants were asked about their least and most favorite activities. They described how they felt about the session content and their thoughts on the curriculum activities. Based on their responses, several participants seemed to enjoy the videos. A few participants commented on how they found the discussions beneficial to understanding the lesson. In relation to the practices taught, mixed responses were received. Many youth reported how they continued to use the practices that they learned. When youth were asked about what factors facilitated their attendance, self-determination and motivation were common themes. Completing the sessions seemed to also provide some participants with a feeling of motivation and a sense of accomplishment. Often youth residing in the shelter were either attending job interviews, counseling sessions, meetings with their case workers, or were employed. To cater to their different schedules, the sessions took place within the shelter and were repeated on various days and times in order to give participants a chance to attend in case they were not available. While this did not emerge in the interviews, it was clearly observed by the study staff and interventionist as a barrier to attendance. The youth also talked about how their experience with the interventionists helped to motivate them as well. Several of the participants reported that the interventionists created a comfortable environment in which they could be themselves.

Regarding the session logistics and their thoughts about the number, time of day, and length of sessions, the responses. Some youth agreed that the length and number of sessions were satisfactory. Overall, the participants appreciated the incentives provided during the sessions. Participants also shared that they enjoyed the food, coffee, and hygiene kits that were provided during the sessions. When participants were asked to share their feedback on how the program could be improved, several responses centered around the length of the baseline and follow-up surveys. Many agreed that the baseline and follow-up surveys were lengthy and had many questions that seemed repetitive yet were acceptable. When asked if they felt the same way about the pre- and post-session surveys, the participants reported the opposite. Many others agreed with this critique. In regard to other aspects of the study, participants were in agreement with each other about keeping everything else the same.

## Discussion

The qualitative data collected from the exit interviews coupled with the quantitative data on feasibility benchmarks suggests overall success and acceptance of delivering *.b4me* in a one-site randomized trial. Feasibility and acceptability benchmarks were surpassed in this pilot and valuable information was gathered about recruitment, implementation, treatment adherence, study retention, assessment data collection, and content acceptability. This was, in part, due to the commitment of the shelter and study staff and the trusting relationship built with participants throughout the study. This allowed the team to reach participants who may have left the shelter using the extensive follow-up strategies developed to support retention including having multiple contacts for each participant and permission to contact them using social media.

Regarding outcome measures, the brief, pre- and post-session assessments were time-consuming despite being limited to only include the priority measures. Because attendees took the assessments at different paces or a participant may have arrived late delaying the start of the lesson, some attendees left before the lesson started. Therefore, the pre/post-session surveys need to be more brief and nondisruptive and be able to be completed in less than 5 min. Strategies that assisted with data completeness of these surveys included having a backup Wi-Fi hotspot to overcome any disruptions in the shelter-based Wi-Fi and having a minimum of three staff assisting with survey completion and classroom management.

Three measures that will need to be adjusted in future studies include self-compassion, risk-seeking, and stress as the measures used did not meet *a priori* benchmarks for reliability. Therefore, these measures will need to be revisited in subsequent studies and their outcomes here should not be interpreted as valid. One solution would be to expand the Perceived Stress Scale to the 10-item verses the four-item scale and identify and test other self-compassion measures with this population. Further, the entire survey will need to be streamlined to reduce the current length as suggested by participant feedback.

While every effort was made to implement the intervention and control arm similarly, external events throughout the duration of the study caused interruptions and changes in the environment during implementation, including the COVID-19 pandemic-related restrictions of access to the shelter and demolition and relocation of the main shelter recruitment site. The pandemic delayed the initiation of the study and changed the *a priori* schedule of structured activities at the shelter. To reduce the spread of the infection within the shelter residents and staff, the shelter restricted outside visitors, causing additional delays in offering the sessions across the intervention and control arms. Additionally, several key shelter staff left during the pandemic. Further, the shelter was demolished and temporary housing was found across town. The space for group activities at the temporary location was much smaller and less ideal than the shelter space. Since youth often had to give up work hours to attend the sessions, it became critical to also provide an incentive for session attendance to offset the cost to the participant in missed work and potential income.

Despite the numerous challenges faced, youth were very interested in participating in the study. It is possible this was enhanced by the receipt of a study-issued phone and incentives for which participants were eligible. While this is an attractive incentive and critical to maintaining contact with a highly transient population, measures needed to be taken to ensure participants fully considered the commitment required for study participation. The step-wise enrollment process enabled staff to give potential participants adequate time to consider study expectations prior to fully enrolling in the study.

The COVID-19 pandemic created numerous challenges to implementing the study procedures and timeline. The study implementation was delayed as the shelter enforced restricted building access to reduce the risk of COVID-19 outbreaks among residents and staff. Coupled with the planned shelter demolition and the subsequent move to a temporary location, the study activities were adjusted several times during the study period. One such adjustment was increasing the number of times a session was offered to allow participants maximum flexibility to participate while also meeting their immediate needs of securing employment and education, keeping their appointments for needed healthcare, and meeting their mental health needs. YEH were often new to the shelter and adjusting to being newly homeless, a new environment, needing to manage appointments with case managers, obtaining essential documents such as IDs, and needing to secure employment or enroll in school. Prior studies in similar populations have found that even with less than full attendance, participants can experience improvements in substance use and sexual risk behavior (Biegel et al., 2009; Himelstein et al., 2012; Tucker et al., 2017). Despite these competing priorities, participant incentives and full shelter collaboration allowed the study to continue successfully reaching the a priori benchmarks for implementation and treatment adherence. This finding reiterates that enhancing flexibility in delivering interventions (e.g., offering many touchpoints, variety of days and times) to populations that experience extreme challenges can improve adherence.

While data collection can be challenging among YEH, with a priori efforts such as comprehensive retention and follow-up procedures and study-issued phones, it is possible to meet a priori benchmarks. Further, extreme and unanticipated challenges, such as the global pandemic and the demolition of the main recruitment and implementation site, can be overcome with strong community partnerships, study team commitment, and trust-building with participants (Santa Maria et al., 2024).

Both the quantitative and qualitative data indicate that *.b4me* is acceptable to YEH who are residing in a shelter. The youth highlighted the importance of the relatability of the videos used and created. They felt that the session discussions were understandable and aligned well with the session lessons. They appreciated the diverse teaching modalities used and the variability of activities provided. Youth thought that the sessions were about the right number and length and that the surveys were reasonable.

## Limitations and future directions

While this pilot resulted in valuable data to inform a randomized control trial, there were several limitations. The COVID-19 pandemic and demolition of the shelter may have impacted adherence to the intervention and retention that we were unable to fully measure. Additionally, due to unforeseen circumstances, two facilitators trained to deliver *.b4me* was unable to continue working with the study. As a result, only one facilitator delivered the vast majority of the intervention sessions while two facilitators delivered the control condition. Another possible limitation was the use of compensation for session attendance. Since attending a session often meant not attending another obligation such as another life skills class or work and it is standard practice that YEH get compensated for the various activities that they participate in at a shelter, this study also provided incentives. This strategy could limit the scalability of the intervention. However, it does approximate universal shelter-based operations.

This one-site, attention control randomized trial demonstrated that an adapted MBI, *.b4me,* is feasible and acceptable among sheltered YEH. We were able to recruit, implement, and retain YEH in the study as designed with minor adjustments to account for the challenges experienced during the pandemic and with the shelter demolition. The strong collaborative partnership with the shelter staff, experienced study staff, flexible intervention delivery schedule, highly engaged youth, and strong support from the mindfulness curriculum creators, allowed for the study to succeed. Given the challenges experienced, future studies are needed to determine the feasibility of conducting a multi-site randomized trial to demonstrate that an adequate sample size can be recruited and retained prior to conducting a fully powered randomized trial to test the efficacy of *.b4me*.

## Data Availability

The raw data supporting the conclusions of this article will be made available by the authors, without undue reservation.
