# Correction: Feasibility of conducting a pilot randomized trial of a mindfulness-based intervention among sheltered young adults experiencing homelessness

**DOI:** 10.3389/fpsyg.2026.1794869

**Published:** 2026-02-16

**Authors:** Diane Santa Maria, Paula Cuccaro, Erica Sibinga, Kimberly Bender, Ethel Jacko, Widumini Liyanage, Jennifer Jones, Stanley Cron

**Affiliations:** 1 Center for Nursing Research, Cizik School of Nursing, University of Texas Health Science Center at Houston, Houston, TX, United States; 2 Health Promotion and Behavioral Sciences, The University of Texas Health Science Center at Houston, Houston, TX, United States; 3 Department of Pediatrics, The Johns Hopkins University School of Medicine, Baltimore, MD, United States; 4 Graduate School of Social Work, University of Denver, Denver, CO, United States

**Keywords:** mindfulness, youth, young adults, homelessness, feasibility

In the published article, it was incorrectly stated that there were no significant differences in baseline characteristics between groups when referring to Table 3.

A correction has been made to the section: **Results**, “*Sample characteristics*,” paragraph #1.

“There were statistical differences in age (*p* = 0.029) and age at first homelessness (*p* = 0.022) in baseline characteristics between groups (Table 3). However, these differences between the groups were not deemed to be practically significant.”

The original version of this article has been updated.

